# Efficacy and safety of canagliflozin in combination with insulin: a double-blind, randomized, placebo-controlled study in Japanese patients with type 2 diabetes mellitus

**DOI:** 10.1186/s12933-016-0407-4

**Published:** 2016-06-18

**Authors:** Nobuya Inagaki, Shin-ichi Harashima, Nobuko Maruyama, Yutaka Kawaguchi, Maki Goda, Hiroaki Iijima

**Affiliations:** Department of Diabetes, Endocrinology and Nutrition, Kyoto University Graduate School of Medicine, Kyoto, Japan; Clinical Research Department II, Mitsubishi Tanabe Pharma Corporation, Tokyo, Japan; Data Science Department, Mitsubishi Tanabe Pharma Corporation, Tokyo, Japan; Medical Science Center, Mitsubishi Tanabe Pharma Corporation, Tokyo, Japan

**Keywords:** Canagliflozin, Combination therapy, Insulin, Japanese patients, SGLT2 inhibitor, Type 2 diabetes mellitus

## Abstract

**Background:**

Combination therapy with canagliflozin and insulin was investigated in a prescribed substudy of the canagliflozin Cardiovascular Assessment Study (CANVAS); however, it was not evaluated in Japanese patients with type 2 diabetes mellitus (T2DM). Since the usage profile of insulin therapy and pathologic features of Japanese patients differ from those of Caucasian patients, we determined the clinical benefit of such a combination therapy in Japanese patients.

**Methods:**

Patients who had inadequate glycemic control despite insulin, diet and exercise therapies were randomized into placebo (*n* = 70) and canagliflozin 100 mg (*n* = 76) groups that were administered once daily in addition to their prior insulin therapy in this double-blind, placebo-controlled study. The primary endpoint was the change in glycated hemoglobin (HbA1c) levels from the baseline to week 16.

**Results:**

There was a statistically significant decrease in HbA1c levels from the baseline in the canagliflozin group (−0.97 ± 0.08 %) compared with the placebo group (0.13 ± 0.08 %) at week 16 [last observation carried forward (LOCF)]. The decrease in HbA1c levels in the canagliflozin group was independent of the insulin regimen (premixed, long-acting and long-acting plus rapid- or short-acting). Compared with the placebo group, canagliflozin significantly decreased fasting plasma glucose levels (−34.1 ± 4.8 vs −1.4 ± 5.0 mg/dL) and body weights (−2.13 ± 0.25 vs 0.24 ± 0.26 %), and significantly increased HDL cholesterol (3.3 ± 1.0 vs −0.5 ± 1.0 mg/dL) and HOMA2- %B (10.15 ± 1.37 vs 0.88 ± 1.42 %). The overall incidence of adverse events was similar between the two groups. The incidence and incidence per subject-year exposure of hypoglycemia (hypoglycemic symptoms and/or decreased blood glucose) were slightly higher in the canagliflozin group (40.0 % and 7.97) than in the placebo group (29.6 % and 4.51). However, hypoglycemic events in both groups were mild in severity and dose-reduction of insulin by <10 % from the baseline following hypoglycemic events decreased the incidence per subject-year exposure in the canagliflozin group. The incidence of hypoglycemia between the groups did not differ according to the insulin regimen.

**Conclusion:**

Canagliflozin in combination with insulin was effective in improving glycemic control and reducing body weight and well tolerated by Japanese patients with T2DM.

*Trial Registration* ClinicalTrials.gov identifier: NCT02220920

**Electronic supplementary material:**

The online version of this article (doi:10.1186/s12933-016-0407-4) contains supplementary material, which is available to authorized users.

## Background

Type 2 diabetes mellitus (T2DM) is a worldwide problem that is growing in prevalence. The International Diabetes Federation estimates that 382 million people had diabetes globally in 2013 and predicts that 592 million people will suffer from the disease in 2035 [1]. Chronic hyperglycemia caused by diabetes is associated with microvascular and macrovascular complications, which deteriorate the quality of life and increase cardiovascular events. Therefore, glycemic control is important to prevent diabetic complications and to maintain quality of life [2].

T2DM is conventionally treated with insulin secretagogues, insulin sensitizers, glucose absorption inhibitors, insulin, and glucagon-like peptide-1 receptor agonists [2, 3]. Intensive glycemic control with insulin therapy prevents diabetic complications [4–6]. However, insulin therapy is associated with the risk of hypoglycemia and weight gain [7–9]. Moreover, weight gain may exacerbate insulin resistance, resulting in the need for an increased dose of insulin, which may cause further weight gain. In addition, the effect of blood glucose, rate of hypoglycemia, and weight gain differ among insulin regimens [10]. Therefore, it is important to determine the insulin regimen according to the patient’s background [3].

Inhibitors of the sodium glucose co-transporter 2 (SGLT2) suppress glucose reabsorption in renal tubules and exert insulin-independent antihyperglycemic effects. In addition, this class of drugs decreases body weight [11, 12]. The SGLT2 inhibitor canagliflozin has been approved for the treatment of T2DM by the regulatory authorities of numerous countries across North America, Europe, Latin America, and Asia–Pacific [13]. The efficacy and safety of canagliflozin monotherapy and in combination with other oral antihyperglycemic agents were demonstrated by studies conducted in Japan [14, 15]. Combination therapy with canagliflozin and insulin was investigated in a prescribed sub study of the canagliflozin Cardiovascular Assessment Study (CANVAS) [16]. However, the effects of a combination of canagliflozin and insulin in Japanese patients with T2DM have not been investigated. The usage profile of insulin therapy and pathologic features of Japanese patients differ from those of Caucasian patients [17–19]. Therefore, it is important to determine the clinical benefit of such a combination therapy in Japanese patients. In the present study, we evaluated the efficacy and safety of canagliflozin in combination with insulin in Japanese patients with T2DM who had inadequate glycemic control despite insulin, diet, and exercise therapies. We further assessed the efficacy and safety of canagliflozin combined with different insulin regimens.

## Methods

### Study design

**Fig. 1:**
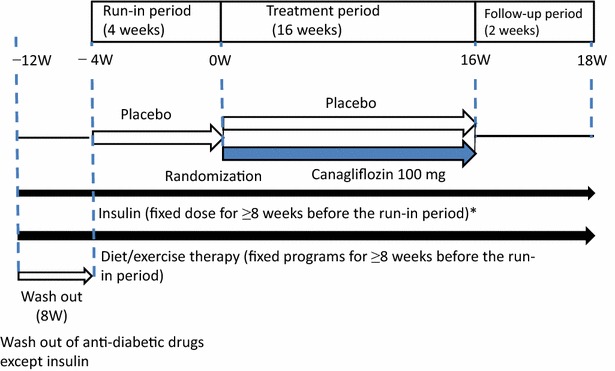
Study design. *Asterisk* accepted when the difference between daily doses of each insulin product and total insulin products were ±10 % of those on the first day of treatment

We conducted a randomized, parallel-group, double-blind study to evaluate the efficacy and safety of canagliflozin in Japanese patients with T2DM who had inadequate glycemic control despite insulin, diet and exercise therapies (Fig. 1). After a 4-week single-blind run-in period, eligible patients were randomized and administered placebo or 100 mg of canagliflozin once daily before breakfast for 16 weeks. Randomization was performed using a block allocation method (1:1, block sizes of 4 and 87 blocks).

The patients received one of the insulin regimens as follows: premixed, intermediate-acting, long-acting, premixed plus rapid- or short-acting, intermediate-acting plus rapid- or short-acting, long-acting plus rapid- or short-acting. The daily dose of insulin ranged from 8 to 60 units. In principle, the insulin dose was fixed during the study period; however, the change within ±10 % of the total daily dose of insulin from the baseline was allowed in order to avoid or treat hypoglycemia or other concomitant illnesses.

### Compliance with the declaration of Helsinki and informed consent

This study was conducted in the spirit of the ethical principles grounded in the declaration of Helsinki and in compliance with Japanese laws related to ensuring drug/medical device quality, efficacy, and safety and Japanese ministerial orders and related regulations on good post-marketing surveillance practice and good clinical practice. The study was approved by the ethics committee/instructional review boards at all of the participating institutions (see List of participating investigators under "Acknowledgements" section). All patients provided written informed consent.

### Inclusion and exclusion criteria

Criteria for including patients were as follows: fixed diet and exercise therapy, receiving a stable dose and regimen of insulin over the 12 weeks before the start of treatment (week 0), glycated hemoglobin (HbA1c) levels of ≥7.5 to <10.5 %, and not taking prohibited antidiabetic drugs during the 12 weeks before week 0. Criteria for excluding patients were as follows: type 1 DM (T1DM), DM caused by a pancreatic disorder, or secondary DM (e.g. Cushing’s syndrome and acromegaly); severe diabetic complications (proliferative diabetic retinopathy, stage 4 nephropathy, or serious diabetic neuropathy); hereditary glucose–galactose malabsorption or primary renal glycosuria; systolic blood pressure of ≥160 mmHg or diastolic blood pressure of ≥100 mmHg; serious renal or hepatic disease; estimated glomerular filtration rate of <45 mL/min/1.73 m^2^; alcoholics; pregnant or possibly pregnant; breastfeeding a child; and refusal to use contraception.

### Outcome measures

The primary endpoint was the change in HbA1c levels from the baseline to week 16 [last observation carried forward (LOCF)]. The secondary endpoints were the changes from the baseline in HbA1c levels at each evaluation point, fasting plasma glucose (FPG), body weight, systolic and diastolic blood pressure, lipids [fasting triglycerides, high-density lipoprotein (HDL) cholesterol], fasting proinsulin/C-peptide ratio, and homeostasis model assessment 2 steady-state beta-cell function (HOMA2- %B). HOMA2- %B was calculated using FPG and fasting C-peptide values. An Excel version of the HOMA calculator of the Diabetes Trial Unit at the University of Oxford was used to calculate HOMA2- %B values.

Safety was assessed based on adverse events, hypoglycemic events, and laboratory test values. AEs were judged by the physicians, and the numbers of affected patients and incidence are listed using MedDRA (Ver. 18.0) system organ class and preferred term. Further, study patients performed self-monitoring of fasting blood glucose at least 3 days each week and when experiencing hypoglycemic symptoms. Low blood glucose without symptoms (≤70 mg/dL) was classified as decreased blood glucose. Hypoglycemic episodes with a typical hypoglycemic symptom were classified as hypoglycemia, regardless of the blood glucose level.

### Statistical analysis

For the primary and secondary endpoints, point estimates of intergroup difference (canagliflozin group − placebo group) in least squares (LS) means were calculated along with the corresponding standard error (SE), 95 % confidence interval, and *p* value. Analysis of covariance (ANCOVA) was performed to determine absolute or percentage changes from the baseline to each evaluation point, with the baseline value as a covariate. Changes in HbA1c levels from the first day of treatment to each evaluation point were analyzed using mixed-model repeated-measures (MMRM) with restricted maximum likelihood. All statistical analyses were conducted using SAS 9.4 (SAS Institute Inc., Cary, NC, USA)

## Results

### Patient disposition and demographic characteristics

Of the 201 patients who consented to participate, 186 entered in the run-in period, and 146 patients were randomized for treatment with placebo (*n* = 70) or canagliflozin (*n* = 76). One patient in the canagliflozin group was mistakenly administered placebo. This patient was included in the canagliflozin group in the full analysis set and in the placebo group in the safety analysis set (Additional file 1: Figure S1).

Table 1 shows patient characteristics of the full analysis set. In the placebo and canagliflozin groups, mean ages were 56.1 and 59.7 years, body weights were 69.68 and 69.95 kg, and durations of T2DM were 12.34 and 15.18 years, respectively. The mean HbA1c levels were 8.85 and 8.89 %, and FPG levels were 169.1 and 169.9 mg/dL in the placebo and canagliflozin groups, respectively (Table 1).

**Table 1:** Patient demographics and baseline characteristics (full analysis set)

	Placebo (N = 70)	Canagliflozin 100 mg (N = 76)
Sex, N (%)		
Male	49 (70.0)	44 (57.9)
Female	21 (30.0)	32 (42.1)
Age (years)		
Mean ± SD	56.1 ± 10.9	59.7 ± 9.4
Duration of diabetes (years)		
Mean ± SD	12.34 ± 8.21	15.18 ± 8.61
Body weight (kg)		
Mean ± SD	69.68 ± 13.13	69.95 ± 13.93
BMI (kg/m^2^)		
Mean ± SD	25.99 ± 4.40	26.88 ± 4.82
Waist circumference (cm)		
Mean ± SD	90.80 ± 10.97	92.93 ± 11.87
Diabetic complications, N (%)		
All	48 (68.6)	50 (65.8)
Retinopathy	26 (37.1)	35 (46.1)
Neuropathy	13 (18.6)	14 (18.4)
Nephropathy	28 (40.0)	31 (40.8)
Nondiabetic complications, N (%)		
Hypertension	40 (57.1)	48 (63.2)
Dyslipidemia	49 (70.0)	63 (82.9)
HbA1c (%)		
Mean ± SD	8.85 ± 0.84	8.89 ± 0.81
Fasting plasma glucose (mg/dL)		
Mean ± SD	169.1 ± 52.6	169.9 ± 44.4
Fasting C-peptide (ng/mL)		
Mean ± SD	1.018 ± 0.776	0.959 ± 0.703
HOMA2- %B (%)		
Mean ± SD	24.18 ± 13.84	22.62 ± 11.24
eGFR (mL/min/1.73 m^2^)		
Mean ± SD	86.1 ± 21.7	83.8 ± 18.4
Daily dose of insulin (unit)		
Mean ± SD	28.1 ± 14.0	31.1 ± 15.1
Daily dose of insulin by insulin regimen (unit)		
Premixed		
N	26	28
Mean ± SD	29.0 ± 11.6	33.1 ± 14.7
Intermediate-acting		
N	0	0
Mean ± SD	–	–
Long-acting		
N	24	24
Mean ± SD	20.9 ± 12.2	20.5 ± 12.3
Premixed + rapid-or short-acting		
N	1	0
Mean ± SD	16.0	–
Intermediate + rapid-or short-acting		
N	0	0
Mean ± SD	–	–
Long-acting + rapid-or short-acting		
N	19	24
Mean ± SD	36.7 ± 14.9	39.5 ± 12.1

*N* number of patients, *BMI* body mass index, *HOMA2*- *%B* homeostasis model assessment 2 steady state beta cell function, *eGFR*, estimated glomerular filtration rate

The mean daily dose of insulin was 28.1 units in the placebo group and 31.1 units in the canagliflozin group, and was not remarkably different between the regimens of the placebo and canagliflozin groups: premixed insulin, 29.0 and 33.1 units; long-acting insulin, 20.9 and 20.5 units; and long-acting and rapid- or short-acting insulin, 36.7 and 39.5 units, respectively. No patient used an intermediate-acting insulin product (Table 1).

### Efficacy

**Fig. 2:**
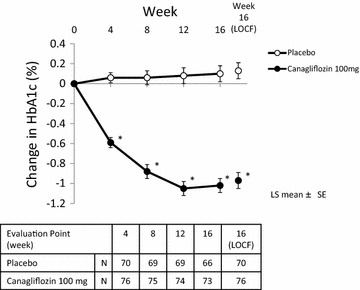
Time course of the change in HbA1c levels from the baseline. Each *point* and *bar* represents LS mean ± SE. *p < 0.001 vs placebo by ANCOVA. The number of patients at each point is shown in the lower table. *N* number of patients at each point, *16 (LOCF)* last observation carried forward to week 16

The changes in HbA1c levels from the baseline at week 16 (LOCF, LS mean ± SE) were 0.13 ± 0.08 % in the placebo group and −0.97 ± 0.08 % in the canagliflozin group, corresponding to the placebo-adjusted changes of −1.10 % (95 % CI, −1.33 to −0.87; *p* < 0.001), which were statistically significant. The MMRM were also statistically significant between the groups (*p* < 0.001), indicating the robustness of the results (Table 2). The statistically significant decrease in HbA1c levels in the canagliflozin group compared with the placebo group were apparent at week 4 and reached a plateau at week 12, which were maintained until week 16 (all; *p* < 0.001) (Fig. 2). The decrease in HbA1c levels in the canagliflozin group was observed independent of the type of insulin regimen (Table 2).

**Table 2:** Effect of canagliflozin on HbA1c levels

	Placebo	Canagliflozin 100 mg
Total		
N	70	76
Mean (SD) baseline (%)	8.85 (0.84)	8.89 (0.81)
LS mean (SE) change (%)^a^	0.13 (0.08)	−0.97 (0.08)
Difference (95 % CI) vs placebo (%)	–	−1.10 (−1.33, −0.87)
*p* value		<0.001
N	66	73
LS mean (SE) change (%)^b^	0.15 (0.08)	−0.98 (0.08)
Difference (95 % CI) vs placebo (%)	–	−1.13 (−1.36, −0.89)
*p* value	–	<0.001
Each insulin regimen		
Premixed		
N	26	28
Mean (SD) baseline (%)	8.70 (0.82)	8.73 (0.73)
LS mean (SE) change (%)^a^	−0.01 (0.13)	−0.89 (0.12)
Difference (95 % CI) vs placebo (%)	–	−0.88 (−1.24, −0.52)
*p* value	–	<0.001
Long-acting		
N	24	24
Mean (SD) baseline (%)	8.89 (0.85)	9.02 (0.87)
LS mean (SE) change (%)^a^	0.26 (0.12)	−1.18 (0.12)
Difference (95 % CI) vs placebo (%)	–	−1.44 (−1.79, −1.09)
*p* value	–	<0.001
Premixed + rapid- or short-acting		
N	1	0
Mean (SD) baseline (%)	7.50 (−)	–
LS mean (SE) change (%)^a^	0.10 (0.00)	–
Long-acting + rapid- or short-acting		
N	19	24
Mean (SD) baseline (%)	9.09 (0.80)	8.96 (0.83)
LS mean (SE) change (%)^a^	0.17 (0.19)	−0.83 (0.17)
Difference (95 % CI) vs placebo (%)	–	−1.00 (−1.51, −0.49)
*p* value	–	<0.001

*N* number of patients, *LS mean* least squares mean, *95* *% CI* 95 % confidence interval

^a^LS mean for change from the baseline to week 16, ANCOVA (*Factor* treatment, *covariate* HbA1C levels at baseline)

^b^LS mean for change from the baseline to week 16, MMRM

**Fig. 3:**
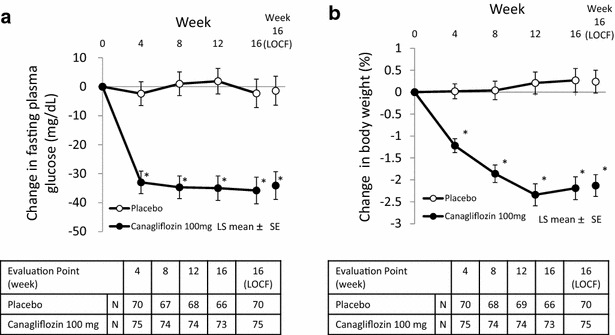
Time courses of the change in (**a**) fasting plasma glucose (FPG) and (**b**) body weight from the baseline. Each *point* and *bar* represents the LS mean ± SE. *p < 0.001 vs placebo by ANCOVA. The number of patients at each point is shown in the lower table. *N* number of patients at each point, *16 (LOCF)* last observation carried forward to week 16

A significant decrease in FPG in the canagliflozin group compared with the placebo group was detected by week 4 and was maintained until week 16 (all; *p* < 0.001) (Fig. 3a). The difference between the canagliflozin and placebo groups regarding the change in FPG (LOCF,LS mean) was −32.6 mg/dL (95 % CI, −46.3 to −18.9; *p* < 0.001) (Table 3).

**Table 3:** Effect of canagliflozin on secondary endpoints

Parameters	Placebo	Canagliflozin 100 mg
FPG (mg/dL)		
N	70	75
Mean (SD) baseline	169.1 (52.6)	170.6 (44.4)
LS mean (SE) change^a^	−1.4 (5.0)	−34.1 (4.8)
Difference (95 % CI) vs placebo	–	−32.6 (−46.3, −18.9)
*p* value	–	<0.001
Body weight (kg)		
N	70	75
Mean (SD) baseline	69.68 (13.13)	70.19 (13.86)
LS mean (SE) change^a^	0.15 (0.18)	−1.49 (0.18)
(%)		
LS mean (SE) percent change^a^	0.24 (0.26)	−2.13 (0.25)
Difference (95 % CI) vs placebo	–	−2.37 (−3.09, −1.65)
*p* value	–	<0.001
SBP (mmHg)		
N	70	76
Mean (SD) baseline	129.95 (16.32)	136.85 (12.01)
LS mean (SE) change^a^	−0.40 (1.19)	−3.58 (1.14)
Difference (95 % CI) vs placebo	–	−3.19 (−6.49, 0.11)
*p* value	–	0.058
DBP (mmHg)		
N	70	76
Mean (SD) baseline	77.23 (10.87)	78.34 (10.18)
LS mean (SE) change^a^	−0.31 (0.74)	−1.55 (0.71)
Difference (95 % CI) vs placebo	–	−1.24 (−3.27, 0.80)
*p* value	–	0.232
Triglyceride (mg/dL)		
N	70	75
Mean (SD) baseline	144.0 (114.0)	124.5 (112.3)
LS mean (SE) change^a^	−4.0 (7.7)	−7.8 (7.4)
Difference (95 % CI) vs placebo	–	−3.8 (−25.0, 17.3)
*p* value	–	0.721
HDL-cholesterol (mg/dL)		
N	70	75
Mean (SD) baseline	57.6 (16.9)	61.9 (16.1)
LS mean (SE) change^a^	−0.5 (1.0)	3.3 (1.0)
Difference (95 % CI) vs placebo	–	3.7 (1.0, 6.5)
*p* value	–	0.007
Proinsulin/C-peptide		
N	69	74
Mean (SD) baseline	0.0267 (0.0323)	0.0235 (0.0380)
LS mean (SE) change^a^	0.0003 (0.0016)	−0.0024 (0.0015)
Difference (95 % CI) vs placebo	–	−0.0026 (−0.0070, 0.0017)
*p* value	–	0.235
HOMA2- %B (%)		
N	69	74
Mean (SD) baseline	24.26 (13.92)	22.23 (11.12)
LS mean (SE) change^a^	0.88 (1.42)	10.15 (1.37)
Difference (95 % CI) vs placebo	–	9.27 (5.35, 13.19)
*p* value	–	<0.001

*N* number of patients, *FPG* fasting plasma glucose, *SBP* systolic blood pressure, *DBP* diastolic blood pressure, *HDL*-*cholesterol* high-density lipoprotein cholesterol, *HOMA2*- *%B* homeostasis model assessment 2 steady state beta cell function, *LS mean* least squares mean, *95* *% CI*, 95 % confidence interval

^a^LS mean for change from the baseline to week 16, (*factor* treatment, *covariate* each parameter at baseline)

The mean body weight of the canagliflozin group significantly decreased from weeks 4 to 12 and was maintained through week 16 (all; *p* < 0.001) (Fig. 3b). The difference between the canagliflozin and the placebo groups regarding the percentage change in body weight from the baseline to week 16 (LOCF, LS mean) was −2.37 % (95 % CI, −3.09 to −1.65; *p* < 0.001) (Table 3).

Other secondary endpoints, including the changes from the baseline to week 16 of systolic and diastolic blood pressures, triglycerides, HDL cholesterol, proinsulin/C-peptide ratio, and HOMA2- %B are summarized in Table 3. The systolic and diastolic blood pressure and triglycerides were decreased from baseline at week 16 in the canagliflozin group; however, there was no significant difference between the canagliflozin and placebo groups. HDL cholesterol was significantly increased in the canagliflozin group compared to the placebo group after 12 weeks of treatment, and the difference between two groups (LOCF, LS mean) was 3.7 mg/dL (95 % CI, 1.0–6.5; *p* = 0.007). The difference between the canagliflozin and placebo groups regarding the change in the fasting proinsulin/C-peptide ratio and HOMA2- %B (LOCF, LS mean), as markers of beta cell function, was −0.0026 (95 % CI, −0.0070 to 0.0017; *p* = 0.235) and 9.27 % (95 % CI, 5.35–13.19; *p* < 0.001), respectively (Table 3).

The insulin doses were increased in 10 patients (14.3 %) and three patients (3.9 %); increased and decreased in one patient (1.4 %) and one patient (1.3 %); and decreased in two patients (2.9 %) and 13 patients (17.1 %) in the placebo and canagliflozin groups, respectively.

### Safety

The overall incidence of adverse events was similar between the two groups (64.8 %, placebo group; 68.0 %, canagliflozin group). The adverse events that occurred more frequently in the canagliflozin group were decreased blood glucose, hypoglycemia, pollakiuria, and polyuria (Table 4). The incidence of hypoglycemia was slightly higher in the canagliflozin group (40.0 %) than in the placebo group (29.6 %). The difference in the incidence ratio between the placebo and canagliflozin groups was 10.4 %, which was not statistically significant (95 % CI, −6.0 to 26.3; *p* = 0.225), and all hypoglycemic events were mild in severity.

**Table 4:** Summary of safety data (safety analysis set)

	Placebo		Canagliflozin 100 mg	
	(N = 71)		(N = 75)	
	n (%)	95 % CI	n (%)	95 % CI
Adverse events	46 (64.8)	52.5–75.8	51 (68.0)	56.2–78.3
Adverse drug reactions	16 (22.5)	13.5–34.0	30 (40.0)	28.9–52.0
Serious adverse events	1 (1.4)	0.0–7.6	3 (4.0)	0.8–11.2
Serious adverse drug reactions	0 (0.0)	0.0–5.1	0 (0.0)	0.0–4.8
Adverse events leading to discontinuation	0 (0.0)	0.0–5.1	1 (1.3)	0.0–7.2
Adverse drug reactions leading to discontinuation	0 (0.0)	0.0–5.1	0 (0.0)	0.0–4.8
Deaths	0 (0.0)	0.0–5.1	0 (0.0)	0.0–4.8
AEs of special interest				
Documented hypoglycemia^a^	21 (29.6)		30 (40.0)	
Hypoglycemia	15 (21.1)		19 (25.3)	
Blood glucose decreased	11 (15.5)		20 (26.7)	
Urinary tract infection	0 (0)		1 (1.3)	
Cystitis	0 (0)		1 (1.3)	
Osmotic diuresis	2 (2.8)		4 (5.3)	
Pollakiuria	1 (1.4)		4 (5.3)	
Polyuria	0 (0)		3 (4.0)	
Thirst	1 (1.4)		1 (1.3)	
Fracture	1 (1.4)		0 (0)	
Foot Fracture	1 (1.4)		0 (0)	
Skin disorder	0 (0)		2 (2.7)	
Seborrheic dermatitis	0 (0)		1 (1.3)	
Urticaria	0 (0)		1 (1.3)	
Ketone bodies	2 (2.8)		3 (4.0)	
Blood ketone bodies increased	2 (2.8)		3 (4.0)	
(Number of female patients)	(N = 22)		(N = 31)	
Vulvovaginitis	0 (0)		1 (3.2)	
Genital candidiasis	0 (0)		1 (3.2)	

MedDRA Ver.18.0 *N* number of patients, *n* number of patients with adverse event,  % = *n*/*N* × 100

^a^Hypoglycemia in the follow-up period was excluded

The mean daily insulin dose during treatment was 29.7 units. Hypoglycemic events occurred similarly in patients receiving lower (<29.7 units), equal, or higher than (≥29.7 units) the average insulin dose. The incidence of hypoglycemia in patients receiving an insulin dose of <29.7 units or ≥29.7 units was 27.5 % (*n* = 11) or 32.3 % (*n* = 10), respectively, in the placebo group and 39.5 % (*n* = 15) or 40.5 % (*n* = 15), respectively, in the canagliflozin group.

The incidence of hypoglycemia and incidence per subject-year exposure did not differ substantially according to the type of insulin regimen received by either the placebo group or the canagliflozin group (Table 5). Additional file 2: Table S1 summarizes the incidence of hypoglycemia at 0:00–5:59, 6:00–11:59, 12:00–17:59, and 18:00–23:59 h. The hypoglycemic events occurred most frequently between 6:00 and 11:59 h. Furthermore, the incidence of hypoglycemia of both groups was not associated with exposure period (data not shown). The incidence of hypoglycemia per subject-year exposure was higher in the canagliflozin group (7.97) than in the placebo group (4.51) (Table 5). For patients whose insulin dose was decreased by the investigator because of a hypoglycemic event, the incidence rate of hypoglycemia per subject-year exposure decreased with dose reduction in the canagliflozin group, regardless of the type of insulin regimen (Table 6).

**Table 5:** Incidence of hypoglycemia classified according to insulin regimen

	Total	Premixed	Long-acting	Premixed + rapid- or short-acting	Long-acting + rapid- or short-acting
Placebo					
Number of patients	N = 71	N = 26	N = 24	N = 1	N = 20
Hypoglycemia *n* (%)	21 (29.6)	6 (23.1)	5 (20.8)	1 (100.0)	9 (45.0)
Cumulative exposure (subject-year)	21.3	7.87	7.25	0.31	5.88
Number of events	96	33	29	3	31
Incidence per subject-year exposure	4.51	4.19	4.00	9.78	5.28
Canagliflozin 100 mg					
Number of patients	N = 75	N = 28	N = 24	–	N = 23
Hypoglycemia n (%)	30 (40.0)	12 (42.9)	8 (33.3)	–	10 (43.5)
Cumulative exposure (subject-year)	22.57	8.35	7.17		7.06
Number of events	180	50	64		66
Incidence per subject-year exposure	7.97	5.99	8.93		9.35

Hypoglycemia in the follow-up period was excluded

*N* number of patients, *n* number of subjects with adverse event,  % = *n*/*N* × 100

**Table 6:** Incidence of hypoglycemia in patients with insulin dose reduction

Type of insulin regimen	Total (N = 3)	Premixed (N = 1)	Long-acting (N = 1)	Premixed + rapid- or short-acting (N = 1)	Long-acting + rapid- or short-acting (N = 0)
Placebo					
Before first dose reduction					
Cumulative exposure (subject-year)	0.72	0.23	0.26	0.23	–
Number of events	16	14	0	2	–
Incidence per subject-year exposure	22.31	60.88	0	8.70	–
After first dose reduction					
Cumulative exposure (subject-year)	0.18	0.08	0.03	0.08	–
Number of events	5	4	0	1	–
Incidence per subject-year exposure	27.26	52.18	0	13.04	–

Type of insulin regimen	Total (N = 14)	Premixed (N = 4)	Long-acting (N = 5)	Premixed + rapid- or short-acting (N = 0)	Long-acting + rapid- or short-acting (N = 5)
Canagliflozin 100 mg					
Before first dose reduction					
Cumulative exposure (subject-year)	1.54	0.42	0.59	–	0.53
Number of events	72	22	20	–	30
Incidence per subject-year exposure	46.88	52.87	33.98	–	56.48
After first dose reduction					
Cumulative exposure (subject-year)	2.55	0.64	0.93	–	0.98
Number of events	62	17	18	–	27
Incidence per subject-year exposure	24.27	26.65	19.28	–	27.47

Hypoglycemia in the follow-up period was excluded. Incidence per subject-year exposure: events/total exposure (subject-year)

*N* number of patients

Adverse events related to osmotic diuresis occurred slightly more frequently in the canagliflozin group than in the placebo group, but adverse events related to volume depletion, which could occur secondarily to osmotic diuresis, were not observed in either group (Table 4).

Serious adverse events were as follows: cataracts (one patient on placebo and one patient on canagliflozin), retinal detachment (one patient on canagliflozin), vitreous hemorrhage (one patient on canagliflozin), and alcoholic liver disease (one patient on canagliflozin). However, a causality assessment of “not related” was assigned to each event. Alcoholic liver disease (in one patient on canagliflozin) resulted in withdrawal from the study.

Small increases in hemoglobin, hematocrit, and blood urea nitrogen levels were detected in the canagliflozin group. AST, ALT and γ-GTP levels were decreased from baseline in the canagliflozin group. The change of LDL-C was not different between placebo and canagliflozin groups. The mean value of the ketone bodies at baseline of both groups was higher than normal range, which was defined as 26.0–122 μmol/L in this study, and the slight increase of the ketone bodies was observed at 16 weeks in canagliflozin group (Additional file 3: Table S2).

## Discussion

### Current findings and implications: efficacy

In the present study, treatment with canagliflozin for 16 weeks improved glycemic control and other metabolic parameters, such as body weight and HDL cholesterol, in Japanese patients with T2DM who received insulin therapy. The decrease in HbA1c levels here was slightly greater than that observed in a previous study in non-Japanese patients, including Caucasians [difference between placebo and canagliflozin (100 mg each) at 18 weeks, −0.62 %] [16], suggesting that the effects of canagliflozin are independent of the pathologic features among races [20]. A significant decrease in HbA1c levels was observed regardless of the type of the insulin regimen.

Administration of insulin to patients with T2DM is often associated with weight gain, but the patients studied here experienced weight loss following combination therapy with canagliflozin and insulin. Similar results were reported by studies on the SGLT2 inhibitors dapagliflozin and empagliflozin used in combination with insulin, which were conducted outside Japan [21–24].

A study on a Japanese population administered a combination therapy of dapagliflozin and insulin demonstrated the improving glycemic control and reducing body weight. However, there are some differences in the present study: about 45 % of the participants were also treated with a dipeptidyl peptidase-4 inhibitor, and the data were not evaluated according to the type of insulin regimen [25]. The results of the present study demonstrated that the combination of canagliflozin and insulin, regardless of the insulin regimen, controlled plasma glucose levels without causing weight gain in Japanese patients with T2DM who were inadequately controled by insulin.

Japanese patients with T2DM tend to have a long duration of disease and have high levels of HbA1c when insulin is initiated [17, 18]. Patients in the present study had a longer duration of DM (approximately 12–15 years) than that of previous studies (approximately 5–8 years in the Japanese phase 3 study) and a higher baseline level of HbA1c [14, 15]. Baseline values of HOMA2- %B and C-peptide were lower in the present study than in those previously reported, which suggests that the patients had a decreased capacity to secrete insulin. Nevertheless, canagliflozin treatment improved glycemic control. These findings are consistent with those of previous studies showing that canagliflozin decreases plasma glucose, regardless of insulin secretory capacity and duration of diabetes mellitus [26, 27]. Interestingly, canagliflozin combination with insulin slightly increased HOMA2- %B, suggesting improved beta-cell function. This is possibly resulting from a reduction of glucotoxicity [12, 28].

### Current findings and implications: Safety

Here the overall incidence of adverse events was similar between the placebo and canagliflozin groups. The incidence of hypoglycemia was slightly higher in the canagliflozin group than in the placebo group. All events were mild in severity, and severe hypoglycemia (i.e., requiring the assistance of another person) was not reported. Hypoglycemic events (hypoglycemic symptoms and/or decreased blood glucose) occurred most frequently at 6:00–11:59 h; therefore, caution may be exercised in the morning for patients who receive the combination of an SGLT2 inhibitor and insulin.

The incidence of hypoglycemia was not markedly different among the types of insulin regimens. In a study on empagliflozin added on to basal insulin, during the first 18 weeks of administration of a fixed insulin dose, the incidence of hypoglycemic events was slightly higher in patients administered 25 mg of empagliflozin than in those administered placebo or 10 mg of empagliflozin. However, after physicians were allowed to titrate the insulin dose, the incidence of hypoglycemia over the complete 78-week treatment was similar among the groups [24]. Similarly, in the present study, the incidence per subject-year exposure decreased in patients undergoing insulin dose reduction following a hypoglycemic event. These findings suggest that adjusting the insulin dose of the combined regimen prevents the occurrence of hypoglycemic events.

The slight increase of the ketone bodies (59.93 μmol/L) from baseline was observed at 16 weeks in canagliflozin group, although it was not notably higher than those reported by previous studies of canagliflozin [14, 15, 29] or other SGLT2 inhibitor [30]. Malaise and similar symptoms that may accompany the marked elevation of ketone bodies were not reported, and no patient was dismissed because of increased blood ketone bodies in this study. The elevation of ketone bodies was not accompanied by hyperglycemia and is therefore likely attributable to a compensatory increase in fatty acid metabolism in response to loss of calories because of canagliflozin-induced urinary glucose excretion.

### Future perspectives

Several clinical studies have reported the safety and efficacy of SGLT2 inhibitors in combination with insulin in patients with T1DM, however diabetic ketoacidosis has been reported in some studies [28, 31–34]. In addition, diabetic ketoacidosis has been reported in patients with T1DM who were treated off-label with an SGLT2 inhibitor in daily clinical practice [35, 36].Therefore application of SGLT2 inhibitors for T1DM still remains to be addressed.

On the other hand, some cases of diabetic ketoacidosis have also been reported in patients with T2DM who were treated with an SGLT2 inhibitor. Lowering the dose of insulin may increase the production of ketone bodies because of insufficient suppression of lipolysis and ketogenesis [35]. Therefore adjusting the insulin dose may be performed with care, particularly in T2DM patients with diminished capacity to secrete insulin.

There were no cardiovascular-related AEs both placebo and canagliflozin group in this study. Several studies of SGLT2 inhibitors for assessment of the cardiovascular outcome are conducting [37], and it was recently reported that the SGLT2 inhibitor empagliflzoin reduces cardiovascular event in T2DM patient with high CVD risk, EMPA-REG OUTCOME trial, around 48 % of subjects were on insulin-combination therapy [38]. In the CANVAS trial, about half of the subjects were also treated with insulin [39]. These studies will provide the information on the effect of the combination of SGLT2 inhibitor and insulin on cardiovascular outcome.

## Limitations of the study

The limitation of this study is the short course of treatment; hence, the present study has been extended for up to 52 weeks. In addition, patients who were treated with insulin in the form of an intermediate-acting or rapid-acting product were not involved, and there were a small number of patients in each type of insulin subgroup. Therefore, we did not discuss which insulin regime fit better with canagliflozin.

## Conclusion

Canagliflozin added to insulin therapy was effective and well tolerated by Japanese patients with T2DM. This regimen provides a novel option in the treatment of patients with T2DM who require additional treatment.

## Additional files

10.1186/s13104-016-2115-2 Flow Diagram and number of subjects in each analysis set.
10.1186/s13104-016-2115-2Time of onset of hypoglycemia (safety analysis set).
10.1186/s13104-016-2115-2 Laboratory variables at baseline and change from baseline on week 16 (safety analysis set).

## Abbreviations

ANCOVA: analysis of covariance
HbA1c: glycated hemoglobin
FPG: fasting plasma glucose
HDL: high-density lipoprotein
LS: least squares
LOCF: last observation carried forward
HOMA2- %B: homeostasis model assessment 2 steady-state beta-cell function
MMRM: mixed-model repeated-measures
SGLT: sodium glucose co-transporter
SE: standard error
T2DM: type 2 diabetes mellitus
