# Supplementary figures and images for: Efficacy and safety of canagliflozin in combination with insulin: a double-blind, randomized, placebo-controlled study in Japanese patients with type 2 diabetes mellitus
Source: Cardiovasc Diabetol. 2016 Jun 18;15:89. doi: 10.1186/s12933-016-0407-4 (PMC4912792; doi:10.1186/s12933-016-0407-4)

## Slide 1
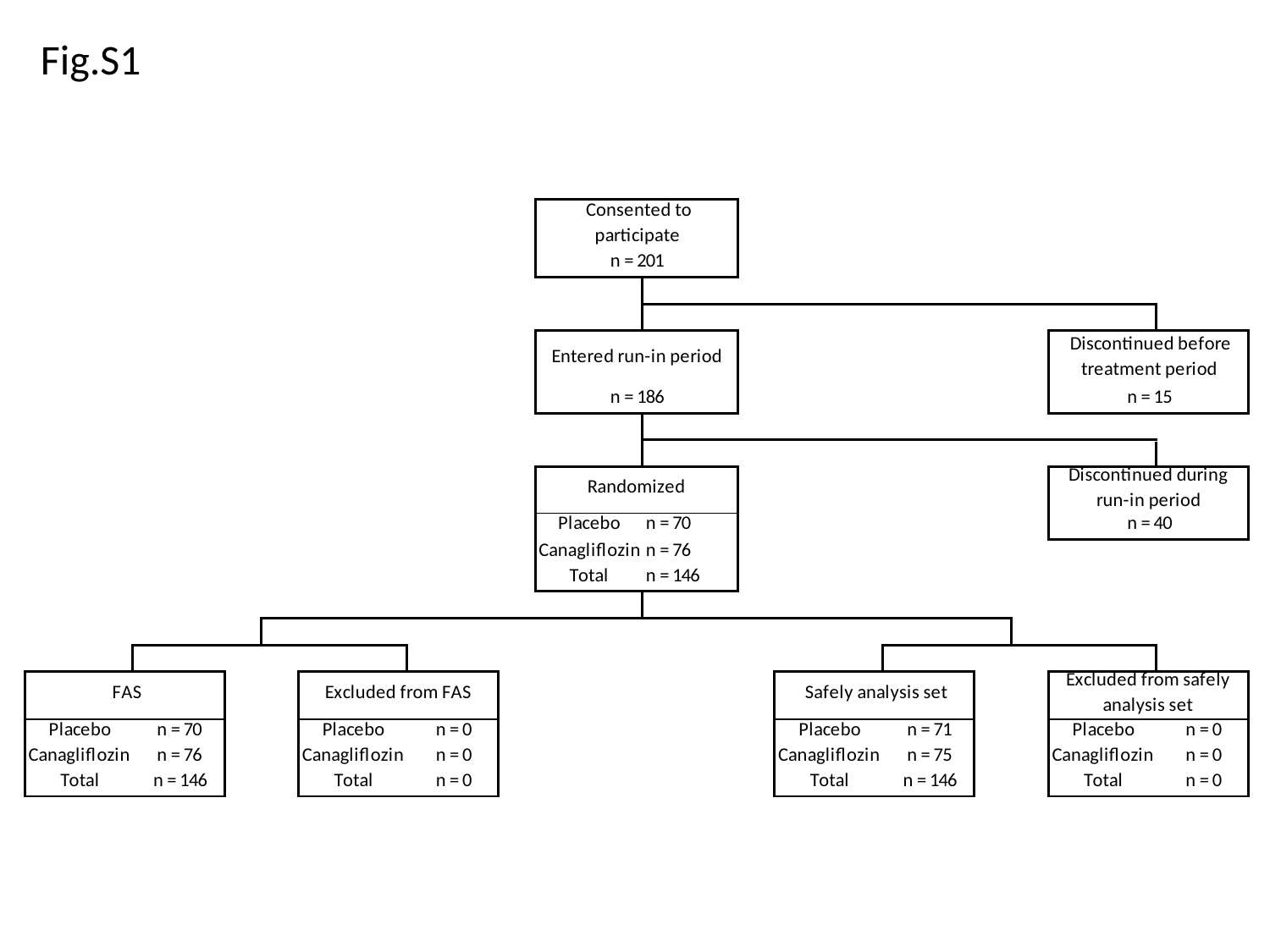

Fig.S1

Supplement: Supplementary file 1 — 10.1186/s13104-016-2115-2 Flow Diagram and number of subjects in each analysis set. [file 12933_2016_407_MOESM1_ESM.pptx]
