# Supplementary material for: Efficacy and safety of canagliflozin in combination with insulin: a double-blind, randomized, placebo-controlled study in Japanese patients with type 2 diabetes mellitus
Source: Cardiovasc Diabetol. 2016 Jun 18;15:89. doi: 10.1186/s12933-016-0407-4 (PMC4912792; doi:10.1186/s12933-016-0407-4)
Supplement: Supplementary file 2 — 10.1186/s13104-016-2115-2Time of onset of hypoglycemia (safety analysis set). [file 12933_2016_407_MOESM2_ESM.docx]

**Table S1 Time of onset of hypoglycemia (Safety analysis set)**

| Time of onset (h) | Placebo | | | | Canagliflozin 100 mg | | | |  |  |  |  |  |  |  |
| --- | --- | --- | --- | --- | --- | --- | --- | --- | --- | --- | --- | --- | --- | --- | --- |
|  | N | n | (%) | Number of events | N | n | (%) | Number of events |  |  |  |  |  |  |  |
| 0:00–5:59 | - | 6 | (8.5) | 8 | - | 11 | (14.7) | 30 |  |  |  |  |  |  |  |
| 6:00–11:59 | - | 12 | (16.9) | 64 | - | 18 | (24.0) | 103 |  |  |  |  |  |  |  |
| 12:00–17:59 | - | 4 | (5.6) | 17 | - | 9 | (12.0) | 24 |  |  |  |  |  |  |  |
| 18:00–23:59 | - | 6 | (8.5) | 7 | - | 8 | (10.7) | 22 |  |  |  |  |  |  |  |
| Total | 71 | 21 | (29.6) | 96 | 75 | 30 | (40.0) | 180 |  |  |  |  |  |  |  |
| *N*, number of patients; *n*, number of patients with adverse event; % = n/N × 100 | | | | | | | | | |  |  |  |  |  |  |
| Hypoglycemia in the follow-up period was excluded | | | | | | | | | | |  |  |  |  |  |
