# Supplementary material for: Efficacy and safety of canagliflozin in combination with insulin: a double-blind, randomized, placebo-controlled study in Japanese patients with type 2 diabetes mellitus
Source: Cardiovasc Diabetol. 2016 Jun 18;15:89. doi: 10.1186/s12933-016-0407-4 (PMC4912792; doi:10.1186/s12933-016-0407-4)
Supplement: Supplementary file 3 — 10.1186/s13104-016-2115-2 Laboratory variables at baseline and change from baseline on week 16 (safety analysis set). [file 12933_2016_407_MOESM3_ESM.docx]

**Table S2 Laboratory variables at baseline and change from baseline on week 16 (Safety analysis set)**

| Parameters |  |  | Placebo | Canagliflozin 100 mg |
| --- | --- | --- | --- | --- |
| Hematocrit | N |  | 66 | 73 |
| (%) | Baseline | Mean (SD) | 43.63 (4.02) | 42.77 (3.57) |
|  | Change from baseline | Mean (SD) | -0.36 (1.77) | 1.93 (2.03) |
| Hemoglobin | |  | 66 | 73 |
| (g/dL) | Baseline | Mean (SD) | 14.64 (1.35) | 14.40 (1.28) |
|  | Change from baseline | Mean (SD) | -0.11 (0.63) | 0.51 (0.67) |
| BUN | N |  | 66 | 73 |
| (mg/dL) | Baseline | Mean (SD) | 14.2 (3.4) | 14.9 (4.2) |
|  | Change from baseline | Mean (SD) | 0.2 (3.0) | 1.6 (4.0) |
| AST | N |  | 65 | 73 |
| (U/L) | Baseline | Mean (SD) | 25.1 (11.2) | 27.3 (11.9) |
|  | Change from baseline | Mean (SD) | -1.2 (9.1) | -3.8 (10.8) |
| ALT | N |  | 66 | 73 |
| (U/L) | Baseline | Mean (SD) | 23.5 (11.7) | 25.9 (19.0) |
|  | Change from baseline | Mean (SD) | -0.8 (9.1) | -5.7 (18.2) |
| γ-GTP | N |  | 66 | 73 |
| (U/L) | Baseline | Mean (SD) | 43.9 (57.7) | 35.0 (29.4) |
|  | Change from baseline | Mean (SD) | 4.4 (34.3) | -7.5 (14.3) |
| Total cholesterol | N |  | 66 | 73 |
| (mg/dL) | Baseline | Mean (SD) | 199.6 (31.4) | 202.1 (36.3) |
|  | Change from baseline | Mean (SD) | 4.8 (27.4) | 6.7 (22.2) |
| LDL-cholesterol | N |  | 66 | 73 |
| (mg/dL) | Baseline | Mean (SD) | 121.9 (27.1) | 122.4 (33.6) |
|  | Change from baseline | Mean (SD) | 4.4 (27.6) | 3.7 (17.8) |
| Total ketone body | N |  | 66 | 73 |
| (μmol/L) | Baseline | Mean (SD) | 135.87 (134.30) | 172.14 (147.19) |
|  | Change from baseline | Mean (SD) | 6.64 (181.66) | 59.93 (235.32) |
| *N*, number of patients; *BUN*, blood urea nitrogen; | | |  |  |
| *AST*, aspartate transaminase; *ALT*, alanine aminotransferase; | | |  |  |
| *γ-GTP*, γ-glutamyl transpeptidase; | |  |  |  |
| *LDL-cholesterol*, Low-density lipoprotein cholesterol. | | | | |
